# 0.9% Sodium chloride solution versus Plasma-Lyte 148 versus compound sodium lacTate solution in children admitted to PICU—a randomized controlled trial (SPLYT-P): study protocol for an intravenous fluid therapy trial

**DOI:** 10.1186/s13063-021-05376-5

**Published:** 2021-07-03

**Authors:** Sainath Raman, Andreas Schibler, Renate Le Marsney, Peter Trnka, Melanie Kennedy, Adrian Mattke, Kristen Gibbons, Luregn J. Schlapbach

**Affiliations:** 1 Paediatric Critical Care Research Group, Child Health Research Centre, The University of Queensland, 62 Graham Street, South Brisbane, QLD 4101 Australia; 2 Paediatric Intensive Care Unit, Queensland Children’s Hospital, South Brisbane, Australia; 3 Paediatric Nephrology, Queensland Children’s Hospital, South Brisbane, Australia; 4 Department of Intensive Care and Neonatology, Children’s Research Center, University Children’s Hospital Zurich, Zurich, Switzerland

**Keywords:** Balanced solutions, Bolus, Child, Critical care, Intravenous fluid therapy

## Abstract

**Background:**

Intravenous fluid therapy represents the most common intervention critically ill patients are exposed to. Hyperchloremia and metabolic acidosis associated with 0.9% sodium chloride have been observed to lead to worse outcomes, including mortality. Balanced solutions, such as Plasma-Lyte 148 and Compound Sodium Lactate, represent potential alternatives but the evidence on optimal fluid choices in critically ill children remains scarce. This study aims to demonstrate whether balanced solutions, when used as intravenous fluid therapy, are able to reduce the incidence of a rise in serum chloride level compared to 0.9% sodium chloride in critically ill children.

**Methods:**

This is a single-centre, open-label randomized controlled trial with parallel 1:1:1 assignment into three groups: 0.9% sodium chloride, Plasma-Lyte 148, and Compound Sodium Lactate solutions for intravenous fluid therapy. The intervention includes both maintenance and bolus fluid therapy. Children aged < 16 years admitted to intensive care and receiving intravenous fluid therapy during the first 4 h of admission are eligible. The primary outcome measure is a ≥ 5mmol/L increase in serum chloride level within 48 h post-randomization. The enrolment target is 480 patients. The main analyses will be *intention-to-treat*.

**Discussion:**

This study tests three types of intravenous fluid therapy in order to compare the risk of hyperchloremia associated with normal saline versus balanced solutions. This pragmatic study is thereby assessing the most common intervention in paediatric critical care.

This is a single-centre open-label study with no blinding at the level of delivery of the intervention. Certain paediatric intensive care unit (PICU) patient groups such as those admitted with a cardiac condition or following a traumatic brain injury are excluded from this study.

**Trial registration:**

The study has received ethical approval (HREC/19/QCHQ/53177: 06/06/2019). It is registered in the Australian New Zealand Clinical Trials Registry (ACTRN12619001244190) from 9th September 2019. Recruitment commenced on 12th November 2019. The primary results manuscript will be published in a peer-reviewed journal.

**Supplementary Information:**

The online version contains supplementary material available at 10.1186/s13063-021-05376-5.

## Administrative information

The order of the items has been modified to group similar items (see http://www.equator-network.org/reporting-guidelines/spirit-2013-statement-defining-standard-protocol-items-for-clinical-trials/).

**Table:** 

Title {1}	0.9% Sodium chloride solution versus Plasma-Lyte 148 versus compound sodium lacTate Solution in children admitted to PICU – a randomised controlled trial – SPLYT-P
Trial registration {2a and 2b}.	The trial is registered in the Australian New Zealand Clinical Trials Registry (ACTRN12619001244190).
Protocol version {3}	Protocol Version 6, dated 29/07/2020
Funding {4}	The study was supported by a grant from the Study, Education and Research Trust Account funding scheme 2019, Children’s Health Queensland, Brisbane, Australia. The funding sources had no involvement in study design, analyses, nor interpretation of the results.
Author details {5a}	1. Dr Sainath Raman^1,2^, MRCPCH, PhD, FCICM 2. Prof. Dr. Andreas Schibler^1,2^, MD, FCICM 3. Renate Le Marsney^1^, BMedSc, MPH 4. Assoc. Prof. Dr. Peter Trnka^3^, MD, FRACP, MSc 5. Melanie Kennedy^1,2^, BNursing 6. Assoc. Prof. Dr. Adrian Mattke^1,2^ MBBS, MD, FRACP, FCICM 7. Assoc. Prof. Dr. Kristen Gibbons^1^, BInfoTech, BMaths (Hons), PhD 8. Assoc. Prof. Dr. Luregn J Schlapbach^1,4^, MD, PhD, FCICM Affiliations: 1. Paediatric Critical Care Research Group, Child Health Research Centre, The University of Queensland, Queensland, Australia 2. Paediatric Intensive Care Unit, Queensland Children’s Hospital, South Brisbane, Australia. 3. Paediatric Nephrology, Queensland Children’s Hospital, South Brisbane, Australia 4. Pediatric and Neonatal Intensive Care Unit, Children’s Research Center, University Children's Hospital Zurich, Zurich, Switzerland
Name and contact information for the trial sponsor {5b}	Ms. Sabine Sand Research Partnership manager Faculty of Medicine The University of Queensland, 288 Herston Rd, Herston, Qld 4006 Australia. email: med.rpms@uq.edu.au
Role of sponsor {5c}	The sponsor (The University of Queensland) and the funding body (Children’s Health Queensland) had no involvement in study design, analyses, nor interpretation of the results.

## Introduction

### Background and rationale {6a}

Intravenous fluid therapy (IVFT) is one of the most common interventions in critically ill patients [1]. Intravenous fluid therapy captures both maintenance as well as bolus fluid administration, and volumes of 100 ml/kg and more in the first days of paediatric intensive care unit (PICU) admission have been reported [2]. 0.9% sodium chloride (NS), available since the 18th century [3], represents the default fluid for IVFT in many services worldwide [4–6]. However, administration of NS has been associated with hyperchloremia and metabolic acidosis [7, 8]. These effects of NS are attributed to its high chloride content with zero strong-ion difference [9]. Compound Sodium Lactate (CSL), containing lower sodium and chloride content, has been available and in use for nearly a century [3]. More recently, Plasma-Lyte 148 (PL) has become available as an alternative balanced fluid (composition closer to plasma) with a higher sodium concentration as compared to CSL (Additional file 1) [10].

Several interventional studies in adult critically ill patients have investigated if balanced IVFT solutions lead to improved clinical outcomes [1]. Two recent trials showed a significant reduction in the need for renal replacement therapy favouring CSL compared to NS [11, 12]. Another randomized controlled trial (RCT), comparing NS to PL, demonstrated no difference in the incidence of new-onset acute kidney injury (AKI) between the groups [13]. A large RCT is underway comparing PL to NS used for both resuscitation and maintenance therapy in critically ill adult patients (NCT02721654).

The evidence to support optimal fluid choice in critically ill children is scarce, and there is a lack of larger trials assessing fluid type in a broad PICU population. A matched retrospective cohort study comparing NS and CSL in patients less than 18 years of age with severe sepsis or septic shock showed no difference in mortality, incidence of acute kidney injury (AKI) and new dialysis or length of stay [14]. Kartha et al., in a randomized study, demonstrated similar clinical and biochemical outcomes between CSL and NS in children with acute diarrhoeal illness [15]. Allen et al. observed a greater increase in serum bicarbonate and more rapid improvement in dehydration scores [16] with CSL compared to NS. Balanced solution in perioperative IVFT in children undergoing brain tumour resection was associated with a lower incidence of chloride increase [17]. Balamuth et al. recently reported on a pilot feasibility RCT in children with sepsis comparing CSL and NS, but the study was not powered for clinical outcomes [18]. Whilst results are awaited from a large RCT in India enrolling 710 children with sepsis comparing balanced solutions to normal saline (NCT02835157), another multicentre RCT in children with sepsis has started recruiting **(**NCT04102371). Whilst these trials are directed at providing high-level evidence for the use of balanced solutions versus 0.9% sodium chloride, they are restricted to specific disease groups and may not be representative of the wider PICU population.

The aim of this study is to demonstrate if, in critically ill children admitted to PICU, balanced solutions (PL and CSL), when compared to 0.9% sodium chloride as the IVFT, reduce the incidence of rise in serum chloride level in the first 48 h post-PICU admission.

### Objectives {7}

1. To determine if balanced solutions cause less chloride rise compared to 0.9% sodium chloride as IVFT in critically ill children in PICU.
2. To determine if balanced solutions cause less renal dysfunction compared to 0.9% sodium chloride as IVFT in critically ill children in PICU.
3. To conduct an IVFT trial in children to inform the design of a larger multicentre study powered for patient-centred primary outcome.

### Trial design {8}

Single-centre, open-label three-arm RCT of children aged birth to < 16 years of age admitted to PICU and treated with IVFT.

## Methods: Participants, interventions and outcomes

### Study setting {9}

Paediatric Intensive Care Unit in Queensland Children’s Hospital, Brisbane, Australia.

### Eligibility criteria {10}

Eligible children are identified on admission to the mixed PICU (Table 1). Included are neonates, infants and children <16 years of age requiring IVFT within the past 4 h, which must be within the first 24 h of PICU admission. The exclusion criteria are patients who require disease-specific protocols for IVFT, certain pre-existing conditions such as cardiac patients, major electrolyte abnormalities, and futility. Cardiac patients are excluded because of the local practice of using minimal maintenance fluids and using albumin 4% as the main bolus fluid type in this patient group. Patients with known pre-existing renal disease are not eligible for recruitment as some of the secondary outcomes specifically investigate the impact of the intervention on renal function. This includes patients admitted to PICU post-renal transplantation.

**Table 1:** Inclusion and exclusion criteria

Group	Criterion	Definition
**Inclusion (determined on admission to PICU)**		
	Age	Birth to < 16 years of age
	Location	Admitted to PICU
	Time frame	New admission to PICU within the last 24 h and has received IVFT for ≤ 4 h in PICU
	Decision—clinician decides that IVFT is required	IVFT—all intravenous fluids (boluses and maintenance fluids but not drug dilutions) administered in PICU
	Biochemistry	Admission sodium > 130 mmol/L (measured on admission or no longer than 48 h before randomization)
**Exclusion**		
	Age	≥ 16 years
	Time frame	Received IVFT for > 4 h in PICU
	Pre-existing conditions	- Admitted for a cardiac condition - Chronic kidney disease
	Disease-specific IVFT protocols	- Traumatic brain injury or at risk of cerebral oedema - Burns - Post-liver transplant - Post-renal transplant - Diabetic ketoacidosis - Oncology patients needing hyperhydration

### Who will take informed consent? {26a}

Where possible, prospective informed consent will then be sought from parents by the research nurse or doctor. Due to the emergency nature of admissions to PICU, it is anticipated that in certain situations, timely informed consent may not be feasible. In these cases, ‘consent to continue’ will be employed. The consent process should be completed by 72 h after randomization. If consent is received after 72 h, this will be recorded as a protocol deviation.

### Additional consent provisions for collection and use of participant data and biological specimens {26b}

Not applicable. No other biological specimens will be collected for storage.

## Interventions

### Explanation for the choice of comparators {6b}

The three arms of the trial are:

1. Control arm—0.9% sodium chloride solution (“normal saline”, NS)
2. Intervention arm 1—Plasma-Lyte 148 (PL)
3. Intervention arm 2—Compound Sodium Lactate (CSL)

### Intervention description {11a}

From the time of randomization onwards, the IVFT of the enrolled patient must be provided as per the allocated arm. IVFT includes both maintenance fluids and any potential bolus fluids during the stay in PICU. Total parenteral nutrition will not be considered IVFT. Drug dilutions are not considered IVFT for the study purpose. If a patient was already on another fluid at the time of randomization, the protocol requires the IVFT to change to the allocated fluid. If clinicians decide to provide another study fluid, or another fluid (such as Albumin 4%), the event will be considered a protocol violation and captured as such.

#### Dose and duration of IVFT

Decisions on the indication, dose and duration of IVFT are made by the treating clinician. This ensures a pragmatic approach. The decision to start and stop maintenance and bolus IVFT is also made by the treating clinician.

#### Other PICU care

An arterial, venous or capillary blood gas with measurement of pH, sodium and chloride will be performed at the time of randomization (if no lab results or blood gas are available in the previous 12 h) and at least every 24 h thereafter until discharge whilst the patient is on IVFT and in PICU. All other treatments such as electrolyte supplementation, medications, dextrose infusion, colloid and blood product administration are as per the local institutional procedures. Routine laboratory monitoring of children receiving IVFT, such as daily blood tests to assess organ dysfunction, will be as directed by the clinician.

### Criteria for discontinuing or modifying allocated interventions {11b}

Discontinuation of intervention fluid will be at the discretion of the attending clinician. The potential reasons for this might be a new change in clinical status that makes the patient meet one of the exclusion criteria or study closure by the site. Participants’ clinical data as per the case report form, with the parent’s permission, will be used for data analysis even after cessation of the intervention. If the serum sodium drops to ≤128 mmol/L, the patient will exit the study.

### Strategies to improve adherence to interventions {11c}

Clinicians and bedside nurse will have ongoing education to improve adherence to the protocols.

### Relevant concomitant care permitted or prohibited during the trial {11d}

Total parenteral nutrition, enteral feeds and fluids, fluid used in drug dilution, fluid used as intravenous flush and blood products (packed red cells, fresh frozen plasma, cryoprecipitate) will not be considered for protocol violations.

### Provisions for post-trial care {30}

Patients will exit the study on discharge from PICU. There will be no further follow-up. There is no planned compensation for participants during the trial.

### Outcomes {12}

The *primary outcome* is a dichotomous variable defined as an increase in serum chloride ≥ 5mmol/L within 48 h from the time of randomization. *Secondary clinical outcomes* include organ dysfunction free survival, new-onset AKI, length of PICU stay, length of hospital stay and PICU free survival (Table 2). New-onset AKI will be calculated using predicted baseline values where a baseline serum creatine value has not been measured [20–22]. *Secondary safety outcomes* focus on adverse events with respect to electrolyte derangements and are censored at 48 h from the time of randomization.

**Table 2:** Definition of outcome measures

Outcome	Criterion	Definition
**Primary**		
Hyperchloremia	Increase in serum chloride ≥ 5 mmol/L	- Difference from baseline level to the highest chloride level measured within 48 h post-randomization - *Baseline level:* measured on blood gas at randomization or using the closest lab or blood gas value up to a maximum of 12 h before randomization - *Highest chloride level:* obtained in the first 48 h post-randomization - Patients will be assumed to have no hyperchloremia if chloride has not been measured in the first 48 h post-randomization
**Secondary (clinical)**		
Organ dysfunction free survival	Survival free of organ dysfunction	- Organ dysfunction defined by Paediatric Logistic Organ Dysfunction 2 Score – PELOD-2) [19] - A PELOD-2 score of > 0 indicates organ dysfunction - Censored at 28 days post-randomization - Assume PELOD-2 is zero at discharge from PICU in survivors - PELOD-2 calculated using the worst values of individual components each day until discharge - If a certain variable (such as creatinine) was not measured on a given day, it is assumed to be normal [19]
Acute kidney injury (AKI)	New-onset AKI	- AKI defined as per KDIGO 2012 criteria using serum creatinine values [20] - Includes AKI within the first 7 days post-randomization if not present on admission. Serum creatinine values measured in the first 7 days post-randomization will be used to assess AKI as per KDIGO 2012 criteria - *Baseline creatinine*: closest serum creatinine value prior to randomization, up to 12 h before randomization - *Predicted baseline creatinine*: for children <1 year, the reported predicted creatinine values by Boer et al. will be used, whilst for children >1 year, the below formula will be employed [21]. Mean creatinine (micromol/L) = − 2.37330 − 12.91367 * log_e_ (age) + 23.93581 * (age)^1/2^ [22] *Presence of AKI on admission*: For those with baseline creatinine: - *B*aseline creatinine ≥ 1.5 times predicted baseline creatinine for those with baseline creatinine For those without baseline creatinine: - Patients will be assumed to have no AKI on admission *New onset AKI*: For those with baseline creatinine: - New-onset AKI will be according to KIDIGO 2012 criteria using follow-up creatinine values. If no follow-up creatinine available, these patients would be deemed to have no AKI For those without baseline creatinine: - Predicted baseline creatinine value will be calculated and assumed to be the baseline value. This value will be compared to the follow-up value (where available) as per KDIGO 2012 criteria. Where no follow-up value is available, these patients would be deemed to have no AKI.
Length of stay	Length of stay	- Length of stay in PICU from randomization to discharge - Length of stay in hospital from randomization to discharge
Survival	PICU free survival	- Censored at 28 days from the post-randomization - Patients will be assumed to be alive once discharged from PICU - For patients who die within 28 days, are discharged to a hospice or for palliative care, this value will be recorded as zero
**Secondary safety outcomes, defined as serum electrolyte/metabolite abnormalities present from randomization to 48 h post-randomization**		
Adverse event	Hyperkalaemia	- Serum potassium > 6.2 mmol/L
	Hypokalaemia	- Serum potassium < 2.5 mmol/L
	Hypercalcaemia	- Serum corrected total calcium > 3.1 mmol/L
	Hypocalcaemia	- Serum corrected total calcium < 1.6 mmol/L
	Hypermagnesaemia	- Serum magnesium > 1.4 mmol/L
	Hyponatraemia	- Serum sodium < 125 mmol/L
	Hyperlactataemia	- Arterial or venous blood gas lactate > 4 mmol/L
	Death in hospital	- Number of deaths

Legend: *PICU* paediatric intensive care unit, *AKI* acute kidney injury, *KDIGO* Kidney Disease: Improving Global Outcomes, *PELOD-2* Paediatric Logistic Organ Dysfunction 2 Score

#### Rationale for the primary outcome

In a retrospective cohort study in 1935 children admitted to a mixed tertiary PICU, Barhight et al. observed 2.3 times increased odds of death (95% confidence interval: 1.03 to 5.21) associated with an increase in chloride level of ≥ 5mmol/L in the first 24 h after PICU admission [23]. The increase in chloride value was defined as the difference between the admission (first level obtained after PICU admission) and the maximum chloride level obtained during the first calendar day [23]. Correspondingly, in a post hoc analysis of the Fluid Expansion of Supportive Therapy Study, Levin et al. demonstrated a 1.65mmol/L (95% CI 0.47 to 2.83) mean change in serum chloride level in children who received a 10-ml/kg bolus of NS or 4% albumin relative to those who did not receive a fluid bolus and this was associated with mortality [24]. Therefore, we have chosen a cutoff of ≥ 5 mmol/L increase in chloride level as our primary outcome.

### Participant timeline {13}

The duration of the allocated IVFT treatment will extend until day 28 or one of the following, whichever occurs first:

- Discharge from PICU, including transfer to another intensive care facility;
- Death;
- Withdrawal of consent; or
- Occurrence of a major adverse event likely related to the intervention.

**Fig. 1:**
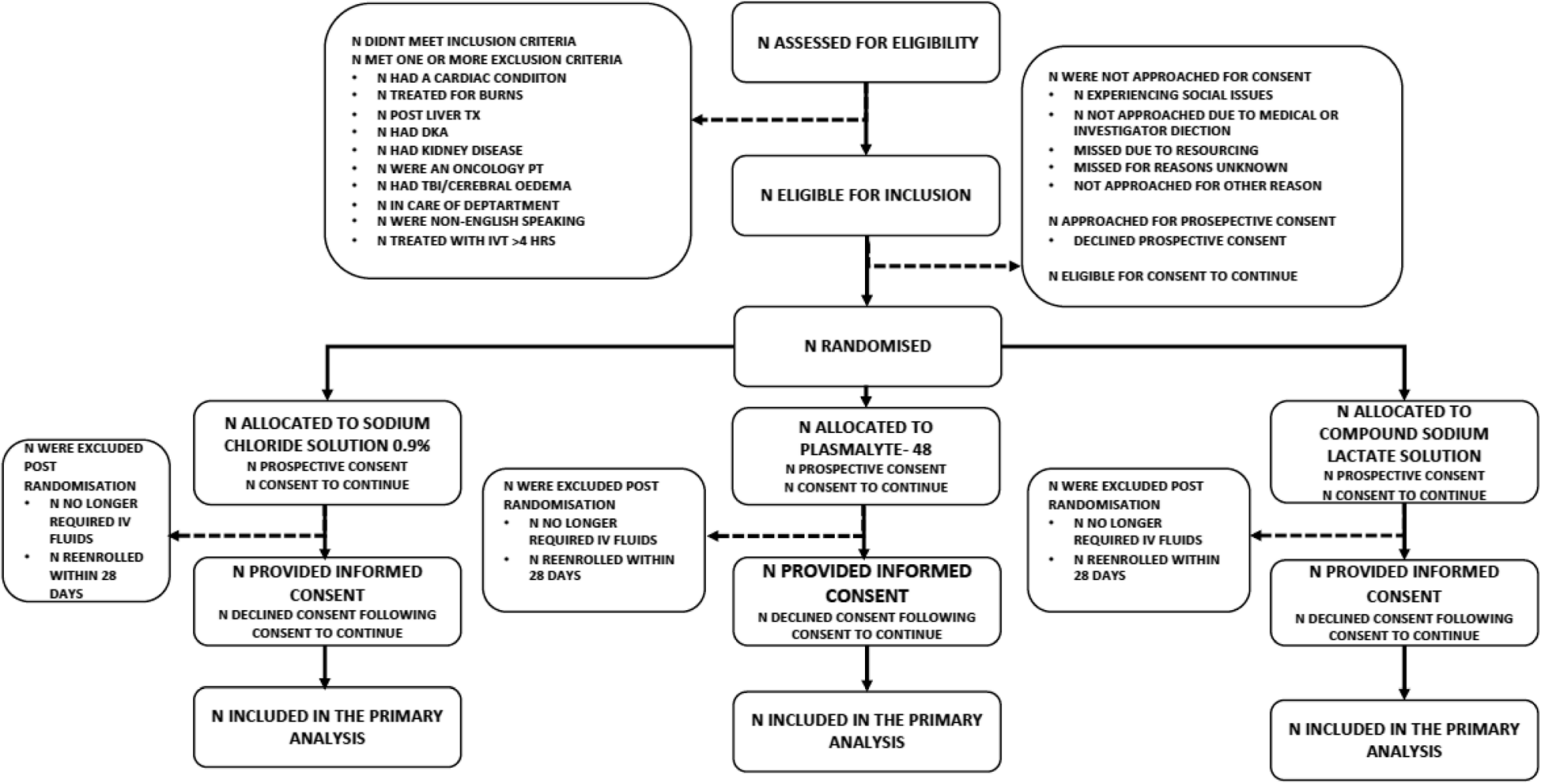
The schedule of enrolment, interventions and assessments

Enrolment, interventions and assessments are outlined in Fig. 1.

### Sample size {14}

The prevalence of a ≥ 5-mmol/L rise in serum chloride level within the first 24 h in children admitted to the PICU has been reported as 12.5% [23]. Based on a review of our institutional PICU data, we expect the proportion of children admitted to PICU requiring IVFT with a ≥ 5-mmol/L rise in chloride level to be approximately 20% by 48 h. To demonstrate a 10% absolute reduction in the prevalence of this primary outcome (setting statistical thresholds of 0.05 for type I error and 80% power), we will need a total of 435 patients. To account for 10% attrition, we will aim to recruit 480 patients. Accounting for ineligible and missed patients, we expect to achieve the recruitment target within an 18-month study period.

### Recruitment {15}

Automated extraction of data items required for screening will be undertaken daily from the electronic medical records of all PICU admissions to create a list of all eligible patients. The research nurse will screen this list to determine eligible patients. On weekdays, the research nurse will screen all patients admitted to the PICU each morning. During on-call hours and weekends, the admitting registrar or consultant intensivist will screen for eligibility for recruitment. The reasons for ineligibility and non-participation of eligible candidates will be documented by the clinical research nurse in the screening log. Co-enrolment with other studies (with different primary outcomes) will be allowed. Where there are repeat admissions to PICU, patients are eligible for re-randomization if the readmission occurs after 28 days of the previous randomization.

## Assignment of interventions: allocation

### Sequence generation {16a}

Online variable block randomization with block sizes of 3, 6 and 9 and equal 1:1:1 allocation into each of the study interventions NS, PL and CSL will be administered through the online REDCap randomization tool hosted by the University of Queensland.

### Concealment mechanism {16b}

REDCap online randomization tool will be employed to implement allocation sequence.

### Implementation {16c}

The allocation sequence will be generated using a computer-based tool and loaded into the REDCap trial database. The bedside nurse or the clinician will enrol patients. REDCap online randomization will assign participants to interventions.

## Assignment of interventions: blinding

### Who will be blinded {17a}

Not applicable. This is an open-label trial.

### Procedure for unblinding if needed {17b}

Not applicable. There is no blinding as part of interventions in this study.

## Data collection and management

### Plans for assessment and collection of outcomes {18a}

Data collection will be performed using a combination of manual data entry into a REDCap study database [25, 26] and automated data extraction from the PICU clinical information system (MetaVisionV 5™, iMDsoft, Tel Aviv) (Additional file 2). Structured query language (SQL) will be used to automate the extraction of the relevant, routinely collected clinical data values from MetaVision^TM^. Information on demographics, the severity on admission, diagnostic codes, interventions and length of stay will be extracted through the mandatory data fields the institution provides to the Australian and New Zealand Paediatric Intensive Care Registry [27]. Further detailed data collection methods are included in Additional file 2 as part of the statistical analysis plan.

### Plans to promote participant retention and complete follow-up {18b}

Participants will remain in the study until discharge from PICU. Data on protocol violations, eligibility and recruitment rate, will be reported. Fluids that are considered as part of the intervention are any fluid bolus and any IV maintenance. Total parenteral nutrition, enteral feeds and fluids, the fluid used in drug dilution, the fluid used as intravenous flush and blood products (packed red cells, fresh frozen plasma, cryoprecipitate) will not be considered for protocol violations. Use of 4% and 20% albumin will be regarded as a protocol violation.

### Data management {19}

Manual primary source verification will be performed in 100% of cases for the inclusion criteria and the primary outcome through independent monitoring staff. Extracted data values for random selected 5% of the study cohort will be manually assessed against the user interface of MetaVision^TM^.

### Confidentiality {27}

Participant confidentiality will be strictly held in trust by the participating investigators, research staff, and the sponsoring institution and their agents. The study protocol, documentation, data and all other information generated will be held in strict confidence. Any data, forms, reports and other records that leave the site will be identified only by a participant identification number (participant ID, PID) to maintain confidentiality. All records will be stored in a secure online trial database. All computer entry and networking programmes will be done using PIDs only. No information concerning the study or the data will be released to any unauthorized third party without the prior written approval of the sponsoring institution. Clinical information will not be released without the written permission of the participant, except as necessary for monitoring by the human research ethics committee or regulatory agencies.

### Plans for collection, laboratory evaluation and storage of biological specimens for genetic or molecular analysis in this trial/future use {33}

Not applicable. No biological samples will be collected for genetic or molecular analysis as part of this trial.

## Statistical methods

### Statistical methods for primary and secondary outcomes {20a}

A detailed statistical analysis plan is available in Additional file 2; the condensed version is presented here. Descriptive statistics will be used to report on the baseline characteristics of the total study cohort, each intervention group and the pre-defined subgroups listed below. The primary outcome will be analysed using multilevel modelling to account for repeated admissions. In the case of a significant result, pre-planned comparisons will be undertaken between NS and each of balanced solutions (i.e., NS versus PL, NS versus CS and PL versus CS, thus three comparisons) and adjusting for multiple comparisons. Secondary outcomes will be analysed using a similar approach, with the specific technique dependent on the distribution of the outcome variable. The primary analyses will be undertaken based on the intention-to-treat (ITT) principle. For ITT, all children who are eligible, randomized, and where consent is available will be included. If the clinician chose not to repeat blood tests whilst in PICU, the data to derive the primary outcome would not be available. In these instances, we will assume that the child was too well for the primary outcome to be met, i.e., the serum chloride level will be assumed not to have increased by ≥ 5 mmol/L within 48 h from randomization. These children will be included in the ITT analyses. We will not impute missing data.

A per-protocol (PP) analysis will also be undertaken for the primary outcome only, including all children who were eligible, randomized, where consent is available, who received the allocated fluid and where both baseline and follow-up chloride measures were obtained.

### Interim analyses {21b}

A formal interim analysis will be performed after the recruitment of 176 children. There are no predetermined stopping rules for this trial.

### Methods for additional analyses (e.g. subgroup analyses) {20b}

We will repeat the ITT and PP analyses for the following subgroups for the primary outcome:

- Age at PICU admission: ≤ 6 months, > 6 months to 5 years, > 5 years to < 16 years
- Admission type: elective versus non-elective admissions
- Patients who received IV fluids for >24 h and ≤ 24 h
- Patients who received > 50 ml/kg IVFT in the first 48 h since randomization and ≤ 50 ml/kg IVFT in the first 48 h since randomization

The age subgroup was chosen to delineate the effect of IVFT on neonates and small infants, young children and adolescents. Children who are non-electively admitted to PICU may be sicker and hence, might receive IVFT for longer. Furthermore, they might have more marked metabolic derangement and respond to IVFT differently to fewer sick patients. Thus, we will be analysing the primary outcome in this subgroup separately. As we hypothesize that the increase in chloride level will be proportional to the amount of intravenous fluid received, we will look at a specific sub-group of patients who received IVFT for > 24 h and those who received > 50 ml/kg (empirical cutoff) in the first 48 h since randomization.

### Methods in analysis to handle protocol non-adherence and any statistical methods to handle missing data {20c}

The proportion of patients who received the allocated fluid will be reported. The proportions of contamination in ml/kg per day for the first 48 h and ml/kg for the entire admission will be reported. Additionally, the number of patients who had baseline chloride and repeat chloride level measured, number of patients who were administered IVFT for > 24 h and who received > 50 ml/kg (empirical cutoff) in the first 48 h since randomization will be reported in total and for each arm. A detailed statistical analysis plan is included in Additional file 2.

### Plans to give access to the full protocol, participant level-data and statistical code {31c}

The authors can provide the full protocol. The statistical analysis plan is attached as Additional file 2. The statistical code is also available at https://github.com/kgibbons44/SPLYTPAnalysis/.

## Oversight and monitoring

### Composition of the coordinating centre and trial steering committee {5d}

SR, LS, MK and KG comprise the steering committee.

### Composition of the data monitoring committee, its role and reporting structure {21a}

Whilst there is not data safety monitoring board for this study, the trial will be overseen by a Trial Steering Committee (TSC), the membership of which will include the PI, one further PI, a senior epidemiologist/statistician and a senior research nurse. The role of the TSC will be to monitor and supervise the progress of the trial and review at regular intervals relevant information from other sources.

### Adverse event reporting and harms {22}

Commonly expected complications related to the underlying disease will not be reported as an adverse event. This may be organ dysfunction such as cardiac or respiratory failure, need for inotropic support or death. Renal dysfunction will be noted separately, as this would be one of the secondary outcomes.

Potential intervention specific adverse events might be hyperkalaemia, hypokalaemia, hypercalcaemia, hypocalcaemia, hypermagnesaemia, hyponatraemia, hyperlactataemia and death in hospital.

Laboratory biochemistry values will be reviewed every day by the attending clinician. Where concerns of an AE exist, this will be discussed with the PI and documented by the clinical research nurse. As these events may be due to the fact that the patient is sick, the attending clinician will be requested to state if, in their personal opinion, the event potentially could be related to the intervention.

For the purposes of this study, the site investigator is responsible for recording all AEs, regardless of their relationship to study intervention, for the period from randomization until discharge from hospital. Conditions that are present at the screening and do not deteriorate will not be considered AEs. AEs will be reported to the PI as soon as possible. The PI, along with the rest of the research team, will review all AEs and determine relatedness and severity. Complications and side effects will be reported using the existing hospital internal reporting structures and databases. The PI will then report back to the patients where appropriate and the ethics committee.

### Frequency and plans for auditing trial conduct {23}

Whilst ongoing regular trial conduct and data monitoring are in place, specific time points, frequency and procedures for formal audits are not planned.

### Plans for communicating important protocol amendments to relevant parties (e.g. trial participants, ethical committees) {25}

All protocol amendments will be approved by the HREC. The ANZPICR application will also be amended accordingly to mirror these changes. The PI and the clinical research nurse will perform ongoing education directed at nurses and doctors. Protocol changes will be highlighted during these sessions.

### Dissemination plans {31a}

Given the lead role of the investigators in paediatric intensive care, their role in regional and state-wide guideline development processes, their involvement in professional colleges and at key educational conferences, it is likely that the findings will have a nationwide impact across Australia and New Zealand.

Publication in high impact peer-reviewed journals will be sought, and presentation at national and international conferences is anticipated. Novel and modern information dissemination strategies will also be used, including social media, podcast presentations and Free Open-Access Medical education (FOAM) resources to generate discussion and disseminate the outcomes of the study.

## Discussion

IVFT is the most common intervention that critically ill children are exposed to. Our trial will be one of the largest randomized controlled trial investigating intravenous fluid management in critically ill children. In addition, given how commonly IVFT is used outside the PICU, trial findings may be directly relevant to children admitted to every hospital.

Our trial is designed as a highly pragmatic trial. Data collection leverages off automated data extraction from the MetaVision^TM^ systems. Whilst making it a robust trial, this methodology has transformed it into a relatively low-cost large randomized control trial. The trial design has the potential to inform future pragmatic trials using electronic health record information.

## Trial status

The current protocol is version 6, dated 29/07/2020. Recruitment commenced on 12th November 2019. The tentative date for the completion of recruitment is 1st May 2021.

## Supplementary Information

**Additional file 1: Table 1.** Composition of three crystalloid solutions compared to plasma.

**Additional file 2 **Statistical analysis plan for 0.9% Sodium chloride solution versus Plasma-Lyte 148 versus compound sodium lacTate Solution in children admitted to PICU (SPLYT-P) Trial.

**Additional file 3.** Parent/Guardian Information Sheet & Consent Form.

## Data Availability

The research team will have access to the trial dataset. Anonymized data could be shared with other investigators for further exploratory analyses after formal approval from the research committee and sponsoring institution.

## Abbreviations

AKI: Acute kidney injury
ATSI: Aboriginal and Torres Strait Islander
CI: Confidence interval
CSL: Compound Sodium Lactate
ED: Emergency department
FFP: Fresh frozen plasma
HFNC: High-flow nasal cannula
ITT: Intention-to-treat
IVFT: Intravenous fluid therapy
KDIGO: Kidney Disease: Improving Global Outcomes
NS: 0.9% sodium chloride
PELOD-2: Paediatric Logistic Organ Dysfunction 2 Score
PICU: Paediatric intensive care unit
PL: Plasma-Lyte 148
PP: Per protocol
RBC: Packed red blood cells
RCT: Randomized controlled trial
SQL: Structured query language
