# Supplementary material for: 0.9% Sodium chloride solution versus Plasma-Lyte 148 versus compound sodium lacTate solution in children admitted to PICU—a randomized controlled trial (SPLYT-P): study protocol for an intravenous fluid therapy trial
Source: Trials. 2021 Jul 3;22:427. doi: 10.1186/s13063-021-05376-5 (PMC8254328; doi:10.1186/s13063-021-05376-5)
Supplement: Supplementary file 1 — Additional file 1: Table 1. Composition of three crystalloid solutions compared to plasma. [file 13063_2021_5376_MOESM1_ESM.docx]

|  | Plasma | 0.9% NaCl solution | Plasma-Lyte 148(1) | Compound Sodium Lactate solution (2) |
| --- | --- | --- | --- | --- |
| Na^+^ (mmol/L) | 140 | 154 | 140 | 131 |
| Cl^-^ (mmol/L) | 100 | 154 | 98 | 112 |
| K^+^ (mmol/L) | 5 | 0 | 5 | 5 |
| Ca^2+^ (mmol/L) | 2.2 | 0 | 0 | 2 |
| Mg^2+^ (mmol/L) | 1 | 0 | 1.5 | 0 |
| Buffer (mmol/L) | 24 | 0 | 50 | 28 |
| Name of buffer | Bicarbonate | N/A | Acetate & Gluconate | Lactate |
| Osmolarity (mOsmol/L) | 275-295 | 308 | 295 | 280 |

Table 1: Composition of three crystalloid solutions compared to plasma

References

1. Reddy SK BM, Beasley RW, Bellomo R, Henderson SJ, Mackle DM, McArthur CJ, Mehrtens JE, Myburgh JA, McGuinness SP, Psirides AJ, Young PJ. . A protocol for the 0.9% saline versus Plasma-Lyte 148 for intensive care fluid therapy (SPLIT) study. Crit Care Resusc. 2014;Dec;16(4):274-9.

2. Semler MW, Kellum JA. Balanced Crystalloid Solutions. American Journal of Respiratory and Critical Care Medicine. 2019;199(8):952-60.
