# Supplementary material for: 0.9% Sodium chloride solution versus Plasma-Lyte 148 versus compound sodium lacTate solution in children admitted to PICU—a randomized controlled trial (SPLYT-P): study protocol for an intravenous fluid therapy trial
Source: Trials. 2021 Jul 3;22:427. doi: 10.1186/s13063-021-05376-5 (PMC8254328; doi:10.1186/s13063-021-05376-5)
Supplement: Supplementary file 2 — Additional file 2 Statistical analysis plan for 0.9% Sodium chloride solution versus Plasma-Lyte 148 versus compound sodium lacTate Solution in children admitted to PICU (SPLYT-P) Trial. [file 13063_2021_5376_MOESM2_ESM.docx]

**Supplementary materials:**

**Statistical analysis plan for 0.9% Sodium chloride solution versus Plasma-Lyte 148 versus compound sodium lacTate Solution in children admitted to PICU (SPLYT-P) Trial**

*Correspondence:*Dr Sainath Raman

62 Graham Street

Paediatric Critical Care Research Group,

Child Health Research Centre, The University of Queensland

South Brisbane 4101

Email: [sainath.raman@uq.edu.au](mailto:sainath.raman@uq.edu.au)

Phone: +61 7 3068 1111

**Statistical analysis principles**

- Analyses will be conducted based on the intention-to-treat principle. Specifically, patients who are eligible, randomised and have valid consent will be analysed based on the treatment group they were allocated to, independent of the compliance with the treatment delivered and whether the patient has come off IVFT and whether serum chloride level was measured again. If serum chloride levels were not measured again after randomisation, it will be assumed that the primary outcome was not met (i.e. no an increase in serum chloride ≥5mmol/L within 48 hours from the time of randomisation).
- A per-protocol analysis will also be undertaken for the primary outcome only. Specifically, patients who are eligible, randomised, have valid consent, who received allocated fluid and both baseline and follow-up chloride measures obtained will be analysed based on the IVFT received, independent of the treatment group they were allocated to.
- Participants with more than one enrolment will have each enrolment included in the analysis of primary and secondary outcomes if the dates of randomisation are greater than 28 days apart.
- The unit of analysis will be the admission rather than the patient.
- Statistical tests will be two-sided applying a statistical significance level of 0.05. Adjustment for multiplicity will not be undertaken when analysing secondary or other outcomes; these results will be considered exploratory and will be reported as point estimates with 95% confidence intervals (CIs).
- No imputation will be undertaken if there are missing data.
- Visual inspection of histograms and Q-Q plots will be used to assess continuous variable for normality.
- Standard descriptive statistics will be used when summarising variables; frequencies (percentages) for discrete variables, mean and standard deviation (SD) for continuous variables, or, if continuous variables are non-normally distributed, median with interquartile range (IQR).
- Pre-planned subgroup analyses will be performed regardless of any potential treatment effect on the primary outcome in the main cohort.
- The Stata (StataCorp Pty Ltd, College Station, Texas) code that will be used to analyse the final study data is available on GitHub (1); this promotes transparency and reliability.
- Changes in the analysis plan by the investigators effective after publication of this statistical analysis plan will be declared as such.

**Data monitoring**

A risk-based data monitoring plan has been devised by the study team based on a risk assessment relating to capture, quality and importance of each data item being collected. This plan was developed in accordance with the ICH E6 (R2) Good Clinical Practice Guideline (2) and implemented the recommendations relating to risk-based monitoring practices.  The following list provides a brief overview of the primary components of the data monitoring plan:

- Data verification on all screening data items (i.e. inclusion and exclusion criteria) on a random sample of 10% of ineligible patients;
- Data verification on all fields imported from the clinical database on 5% of patients;
- Data verification on all screening data items (i.e. inclusion and exclusion criteria) for every enrolled patient;
- Data verification for consent and data items related to calculation of the primary outcome and secondary outcomes that are not imported from the clinical database for every enrolled patient; and
- Source data review on a random sample of 10% of enrolled patients to determine if all adverse events and protocol deviations have been reported.

The original study REDCap database was enhanced to facilitate the data monitoring plan. Data is being monitored throughout the course of the study by a research co-ordinator who is not involved in the study. If a discrepancy is found, the study research co-ordinator and monitor meet to discuss and resolve the discrepancy. If the issue can not be resolved, the principle investigator (SR) is involved. Once data monitoring is finalised, the patient’s REDCap data entry record will be locked in preparation for analysis.

*Interim analyses*

A pre-planned interim analysis was performed after the primary outcome measure was finalised for 176 patients. The interim analysis detailed the primary outcome between treatment groups as well as information on recruitment and adverse events and was reviewed by the Trial Steering Committee. The Haybittle-Peto rule was applied (i.e. a significant p-value less than 0.001 was deemed necessary to warrant consideration of stopping the study early for benefit) (3, 4). The Trial Steering Committee recommended continuation of the trial. The type I error for the final study analyses has not been adjusted to allow for interim analysis.

**Datasets analysed**

The Consolidated Standards of Reporting Trials (CONSORT) flow diagram will be presented based on all patients who were screened for the study (5). All other primary analyses will be performed on the ITT or PP population. If consent is not obtained or withdrawn, data will be excluded from the analyses, unless withdrawn patients permitted the use of data up to the point of withdrawal. The primary dataset for analysis will include baseline variables, PICU admission details, treatment details, outcomes, adverse events and protocol deviations. Following completion of the data monitoring process, data and associated data transformation code will be extracted from the study REDCap database in a Stata (StataCorp Pty Ltd, College Station, Texas) format.

**Data sources**

Data will be entered into a secure web-based trial database, hosted by the University of Queensland. Numerous data sources will be utilised:

| **Data source** | **Variable** | **Mode of extraction** |
| --- | --- | --- |
| MetaVision^TM^ (iMDSoft, Tel Aviv, Israel) | - Screening variables - Serum chloride level before randomisation - Biochemical values before randomisation - Date of admission - Date of discharge | Manually reviewed and entered into the REDCap trial database |
|  | - Physiological variables - Maximum chloride in PICU - Pediatric Logistic Organ Dysfunction Score variables - Intravenous fluids administered from PICU admission to discharge - Organ support - Parameters for assessment of Acute Kidney Injury - Protocol deviations - Adverse events | Automated extraction from MetaVision and imported into REDCap trial database |
| Integrated electronic medical record | - Intravenous fluid administered before admisission to PICU - Biochemical values before admission to PICU | Manually reviewed and entered into the REDCap trial database |
| Australian and New Zealand Paediatric Intensive Care (ANZPIC) Registry | - Demographics - Presenting diagnosis - PICU length of stay - Hospital length of stay | Sourced from registry and imported into REDCap trial database |

**Trial profile and overview**

Recruitment of patients into the trial will be represented using a flow chart based on the CONSORT guidelines (Figure 1). This will describe screened patients, those meeting exclusion criteria, eligible patients, consent process, and those randomised into each of the study arms, with the documentation of the respective primary outcome. We will report on the start and stop date of the trial and provide the recruitment graph by month.

**Patient baseline characteristics**

Baseline characteristics at time of randomisation will be reported for each of the three treatment groups (statistical comparison between groups will not be undertaken) (Table 1).

**Intervention characteristics**

We will provide details on the characteristics of fluid administration by treatment group (Table 2). We will compare characteristics of the procedure that in principle may be altered by the treatment (for example, duration of study fluid, volume of total IV fluid) between the study arms by presenting descriptive statistics along with estimate of difference and 95% confidence interval (CI).

**Outcome measures analysis**

*Primary outcome measure*

The primary outcome measure (≥5mmol/L increase in serum chloride level within 48 hours of randomisation) will be analysed using logistic regression adjusting for treatment group as a fixed effect and patient as a random effects (allowing for the same patient to be recruited more than once into the study), with odds ratios (ORs) and 95% confidence intervals (CIs) reported (Table 3). Assumptions of the model (specification, goodness-of-fit, absence of multicollinearity and absence of influential observations) will be tested and reported on.

*Secondary outcome measures (clinical)*

Binary outcome measures (e.g. new onset AKI) will be treated in the same manner as the primary outcome, without calculation of the p-value. Similar analyses will be undertaken for continuous outcomes; regression with adjustment for treatment group as a fixed effect and patient as a random effect, with reporting of mean difference and 95% CIs. Survival outcomes (length of PICU stay, length of hospital stay) will be visually presented using a Kaplan-Meier plot and a Cox proportional hazard model will be used to assess differences between treatment groups with treatment group as a fixed effect and patient as a random effects (i.e. utilising a shared frailty model). The hazard ratio and 95% CI will be presented as an estimate of treatment effect. Key assumptions of the models (for logistic regression: as per the primary outcome; for survival analysis: proportionality assumption, goodness-of-fit; for linear regression: specification, distribution of residuals, homoscedasticity, absence of multicollinearity, linearity) will be tested and reported on.

*Secondary outcome measures (safety)*

Adverse events will be treated in the same manner as the primary outcome, without calculation of the p-value, using the admission as the unit of analysis. Additionally, the proportion of patients with at least one adverse event will be compared between the three treatment groups using logistic regression as described above for secondary outcomes.

*Protocol Deviations*

Protocol deviations as defined in the study protocol will be presented (Table 5). Additionally, the proportion of patients with at least one protocol deviation will be presented. Statistical comparison between groups will not be undertaken.

*Subgroup and sensitivity analyses*

We will undertake four subgroup analyses on the primary outcome:

- Age at PICU admission: ≤6 months, >6 months to 5 years, >5 years to <16 years;
- Admission type: elective versus non-elective admissions;
- Patients who received IV fluids for: >24 hours, ≤24 hours; and
- Patients who received: >50ml/kg IVFT in the first 48 hours since randomisation, ≤50ml/kg IVFT in the first 48 hours since randomisation.

Subgroup analyses will be undertaken using the same analysis methods described for the primary outcome measure, with the addition of the subgroup variable and its related interaction term into the regression model. For each subgroup, the relevant descriptive statistics will be presented for the primary outcome, along with the appropriate measure of effect size (and 95% CI) and interaction effect (and 95% CI and p-value). A Forest plot will be developed to present heterogeneity between the treatment group and subgroup variable, including the p-value, and presented as a supplementary figure.

**List of supplementary materials:**

1. **Recruitment graph by time**
2. **Kaplan-Meier survival curves**
3. **Forest plot of interaction effects**
4. **Use of blood products**

**REFERENCES**

1. Gibbons K, Marsney R. 0.9% Sodium chloride solution versus Plasma-Lyte 148 versus compound sodium lacTate Solution in children admitted to PICU – a randomised controlled trial: Statistical Analysis Code. 2020 [Available from: <https://github.com/kgibbons44/SPLYTPAnalysis/>].

2. International Council for Harmonisation of Technical Requirements for Pharmaceuticals for Human Use (ICH). ICH Harmonised Guideline. Integrated Addendum to ICH E6(R1): Guideline for Good Clinical Practice E6(R2). [Internet]. 2016.

3. Haybittle JL. Repeated assessment of results in clinical trials of cancer treatment. Br J Radiol. 1971;44(526):793-7.

4. Peto R, Pike MC, Armitage P, Breslow NE, Cox DR, Howard SV, et al. Design and analysis of randomized clinical trials requiring prolonged observation of each patient. I. Introduction and design. Br J Cancer. 1976;34(6):585-612.

5. Moher D, Schulz KF, Altman DG. The CONSORT statement: revised recommendations for improving the quality of reports of parallel-group randomised trials. The Lancet. 2001;357(9263):1191-4.

Table showing the chronology of protocol revisions:

| **Date of change** | **Summary of changes** |
| --- | --- |
| **30.05.2019** | Informed consent (changed from waiver) |
| **23.07.2019** | 1. Individual randomisation instead of cluster crossover design  2. Outcome - Increase in serum chloride level ≥5mmol/L from PICU admission to the highest chloride level within 48 hours of PICU admission  3. Power calculation for outcome added |
| **19.08.2019** | 1. Clarification of the objectives  2. Amendments to background section  3. Additions to secondary (change in strong-ion difference) and safety outcomes (metabolic acidosis and hypokalaemia)  4. Amendments to inclusion and exclusion criteria  5. Addition of stopping rule |
| **21.11.2019** | Amendments to inclusion and exclusion criteria |
| **30.12.2019** | 1. Addition of intervention (blood gas)  2. Amendments to secondary outcomes  3. Addition of time frame for consent to be obtained |
| **29.07.2020** | 1. Amendments to definition of baseline chloride and adverse events  2. Clarification of inclusion age criteria  3.Change in start time for primary outcome |

Figure 1. Draft CONSORT participant flow diagram


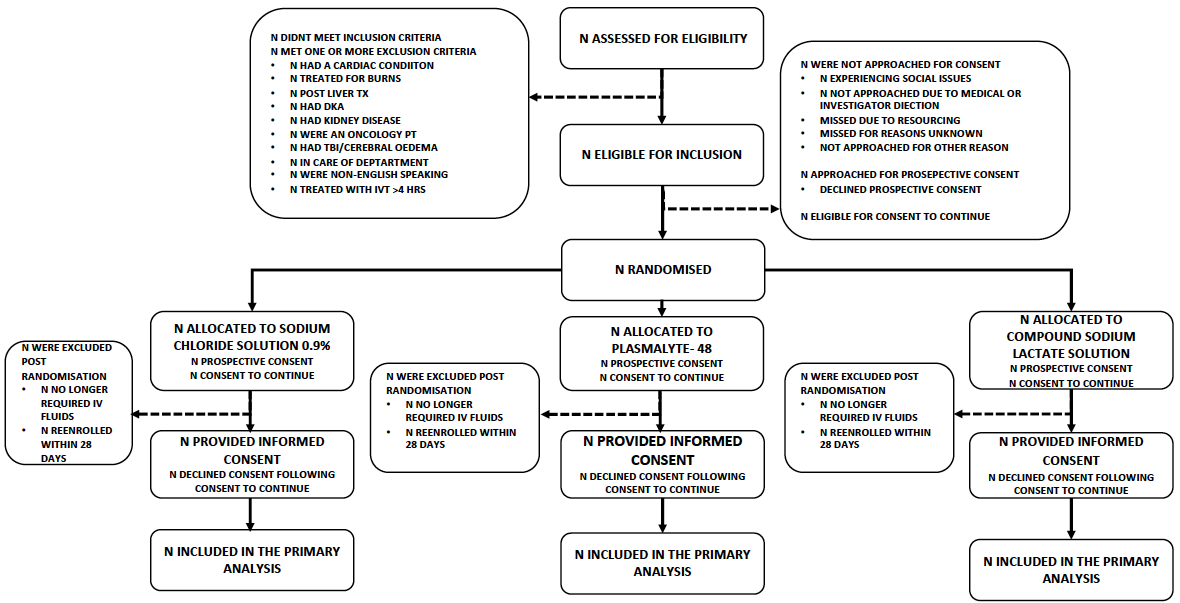


**Table 1: Baseline characteristics**

| **Characteristic** | **0.9% Sodium Chloride**  **N=** | **Plasmalyte-148**  **N=** | **Compound Sodium Lactate**  **N=** |
| --- | --- | --- | --- |
| **Age (year)** *mean (SD)/median (IQR)* |  |  |  |
| **Weight (kg)** *mean (SD)/median (IQR)* |  |  |  |
| **Aboriginal and Torres Strait Islander Status** *n (%)* |  |  |  |
| **Sex, Male** *n (%)* |  |  |  |
| **Presenting diagnosis** |  |  |  |
| Elective admission *n (%)* |  |  |  |
| Post-ENT surgery *n (%)* |  |  |  |
| Post-general surgery *n (%)* |  |  |  |
| Post-other surgery *n (%)* |  |  |  |
| Others *n (%)* |  |  |  |
| Non-elective admission *n (%)* |  |  |  |
| Respiratory infection *n (%)*  Sepsis *n (%)* |  |  |  |
| Oncology *n (%)* |  |  |  |
| Neurological disorders *n (%)* |  |  |  |
| Trauma *n (%)* |  |  |  |
| Others *n (%)* |  |  |  |
| **Co-morbidities** |  |  |  |
| Any comorbidity *n (%)* |  |  |  |
| Syndromes *n (%)* |  |  |  |
| Chronic respiratory disorders *n (%)* |  |  |  |
| Chronic neurological disorders *n (%)* |  |  |  |
| Prematurity *n (%)* |  |  |  |
| Oncology *n (%)* |  |  |  |
| **Admission source** |  |  |  |
| Emergency Department *n (%)* |  |  |  |
| Theatre *n (%)* |  |  |  |
| Hospital ward *n (%)* |  |  |  |
| Retrieved from other hospital *n (%)* |  |  |  |
| Other *n (%)* |  |  |  |
| **Biochemical values on admission** |  |  |  |
| Chloride *mean (SD)/median (IQR)* |  |  |  |
| Sodium *mean (SD)/median (IQR)* |  |  |  |
| Potassium *mean (SD)/median (IQR)* |  |  |  |
| Calcium *mean (SD)/median (IQR)* |  |  |  |
| Creatinine (serum only) *mean (SD)/median (IQR)* |  |  |  |
| **Paediatric Index of Mortality-3** *mean (SD)/median (IQR)* |  |  |  |
| **Organ dysfunction (PELOD-2) on admission** *mean (SD)/median (IQR)* |  |  |  |
| **Mechanical ventilation*** *n (%)* |  |  |  |
| **Cardiovascular support in the first hour#** *n (%)* |  |  |  |
| **Intravenous fluids received prior to randomisation** |  |  |  |
| Volume (ml/kg) *mean (SD)/median (IQR)* |  |  |  |
| Total boluses *mean (SD)/median (IQR)* |  |  |  |
| Total maintenance *mean (SD)/median (IQR)* |  |  |  |
| 0.9% Sodium Chloride *mean (SD)/median (IQR)* |  |  |  |
| Plasmalyte-148 *mean (SD)/median (IQR)* |  |  |  |
| Compound Sodium Lactate *mean (SD)/median (IQR)* |  |  |  |
| Albumin 4% *mean (SD)/median (IQR)* |  |  |  |
| Albumin 20% *mean (SD)/median (IQR)* |  |  |  |

SD standard deviation; IQR interquartile range; ENT ear, nose and throat; PELOD-2 Paediatric Logistic Organ Dysfunction-2

* defined as invasive or non-invasive, excluding high-flow support,

# defined as use of inotropes or vasopressors

Bicohemical variable – measured up to 12 hours before randomisation, PELOD variables – measured closest to admission – one hour prior to one hour after admission.

**Table 2: Description of intravenous fluid therapy administered to randomised patients from randomisation onwards.**

| **Characteristic** | **0.9% Sodium Chloride**  **N=** | **Plasmalyte-148**  **N=** | **Odds ratio (NS vs PL)* (95% CI)** | **Compound Sodium Lactate**  **N=** | **Odds ratio (NS vs CSL)* (95% CI)** |
| --- | --- | --- | --- | --- | --- |
| ***First 24 hours post-randomisation*** | | | | | |
| **All intravenous fluids received** |  |  |  |  |  |
| Duration (hours) *mean (SD)/median (IQR)* |  |  |  |  |  |
| Volume (ml/kg) |  |  |  |  |  |
| Total boluses *mean (SD)/median (IQR)* |  |  |  |  |  |
| Total maintenance *mean (SD)/median (IQR)* |  |  |  |  |  |
| 0.9% Sodium Chloride *mean (SD)/median (IQR)* |  |  |  |  |  |
| Plasmalyte-148 *mean (SD)/median (IQR)* |  |  |  |  |  |
| Compound Sodium Lactate *mean (SD)/median (IQR)* |  |  |  |  |  |
| Albumin 4% *mean (SD)/median (IQR)* |  |  |  |  |  |
| Albumin 20% *mean (SD)/median (IQR)* |  |  |  |  |  |
| **Total allocated fluid** |  |  |  |  |  |
| Duration (hours) *mean (SD)/median (IQR)* |  |  |  |  |  |
| Volume (ml/kg) *mean (SD)/median (IQR)* |  |  |  |  |  |
| Total boluses *mean (SD)/median (IQR)* |  |  |  |  |  |
| Total maintenance *mean (SD)/median (IQR)* |  |  |  |  |  |
| ***First 48 hours post-randomisation*** | | | | | |
| **All intravenous fluids received** |  |  |  |  |  |
| Duration (hours) *mean (SD)/median (IQR)* |  |  |  |  |  |
| Volume (ml/kg) |  |  |  |  |  |
| Total boluses *mean (SD)/median (IQR)* |  |  |  |  |  |
| Total maintenance *mean (SD)/median (IQR)* |  |  |  |  |  |
| 0.9% Sodium Chloride *mean (SD)/median (IQR)* |  |  |  |  |  |
| Plasmalyte-148 *mean (SD)/median (IQR)* |  |  |  |  |  |
| Compound Sodium Lactate *mean (SD)/median (IQR)* |  |  |  |  |  |
| Albumin 4% *mean (SD)/median (IQR)* |  |  |  |  |  |
| Albumin 20% *mean (SD)/median (IQR)* |  |  |  |  |  |
| **Total allocated fluid** |  |  |  |  |  |
| Duration (hours) *mean (SD)/median (IQR)* |  |  |  |  |  |
| Volume (ml/kg) *mean (SD)/median (IQR)* |  |  |  |  |  |
| Total boluses *mean (SD)/median (IQR)* |  |  |  |  |  |
| Total maintenance *mean (SD)/median (IQR)* |  |  |  |  |  |
| ***From randomisation until PICU discharge*** | | | | | |
| **All intravenous fluids received** |  |  |  |  |  |
| Duration (hours) *mean (SD)/median (IQR)* |  |  |  |  |  |
| Volume (ml/kg) |  |  |  |  |  |
| Total boluses *mean (SD)/median (IQR)* |  |  |  |  |  |
| Total maintenance *mean (SD)/median (IQR)* |  |  |  |  |  |
| 0.9% Sodium Chloride *mean (SD)/median (IQR)* |  |  |  |  |  |
| Plasmalyte-148 *mean (SD)/median (IQR)* |  |  |  |  |  |
| Compound Sodium Lactate *mean (SD)/median (IQR)* |  |  |  |  |  |
| Albumin 4% *mean (SD)/median (IQR)* |  |  |  |  |  |
| Albumin 20% *mean (SD)/median (IQR)* |  |  |  |  |  |
| **Total allocated fluid** |  |  |  |  |  |
| Duration (hours) *mean (SD)/median (IQR)* |  |  |  |  |  |
| Volume (ml/kg) *mean (SD)/median (IQR)* |  |  |  |  |  |
| Total boluses *mean (SD)/median (IQR)* |  |  |  |  |  |
| Total maintenance *mean (SD)/median (IQR)* |  |  |  |  |  |

SD standard deviation; IQR interquartile range

* adjusted for patient to account for readmissions

Note: Fluid administered excludes drug dilution, fluids given at a rate of <=2ml/hour, Total parenteral nutrition, and oral fluids. Fluids that are considered as part of the intervention are: any fluid bolus and any IV maintenance. Total parenteral nutrition, enteral feeds and fluids, fluid used in drug dilution, fluid used as intravenous flush and blood products (packed red cells, fresh frozen plasma, cryoprecipitate) will not be considered for protocol violations.

**Table 3: Primary outcome as per intention-to-treat and per-protocol principles in each of the study arms; and subgroups.**

| **Characteristic** | **0.9% Sodium Chloride**  **N=** | **Plasmalyte-148**  **N=** | **Odds ratio (NS vs PL)* (95% CI)** | **p value** | **Compound Sodium Lactate**  **N=** | **Odds ratio (NS vs CSL)*  (95% CI)** | **p value** |
| --- | --- | --- | --- | --- | --- | --- | --- |
| **Total trial cohort: intention-to-treat** |  |  |  |  |  |  |  |
| Increase in serum chloride by ≥5mmol/l during the first 48hours *n (%)* |  |  |  |  |  |  |  |
| **Total trial cohort: per-protocol^#^** |  |  |  |  |  |  |  |
| Increase in serum chloride by ≥5mmol/l during the first 48hours *n (%)* |  |  |  |  |  |  |  |
| **Subgroup: age group^** |  |  |  |  |  |  |  |
| ≤6 months *n (%)* |  |  |  |  |  |  |  |
| >6 months – 5 years *n (%)* |  |  |  |  |  |  |  |
| >5 years – 16 years *n (%)* |  |  |  |  |  |  |  |
| **Subgroup: admission type^** |  |  |  |  |  |  |  |
| Elective *n (%)* |  |  |  |  |  |  |  |
| Non-elective *n (%)* |  |  |  |  |  |  |  |
| **Subgroup: duration of IVFT^** |  |  |  |  |  |  |  |
| ≤24 hours *n (%)* |  |  |  |  |  |  |  |
| >24 hours *n (%)* |  |  |  |  |  |  |  |
| **Subgroup: volume of IVFT^** |  |  |  |  |  |  |  |
| ≤50ml/kg up to 48 hours from randomisation *n (%)* |  |  |  |  |  |  |  |
| >50ml/kg up to 48 hours from randomisation *n (%)* |  |  |  |  |  |  |  |

NS 0.9% Sodium Chloride (Normal Saline); PL Plasmalyte-148; CSL Compound Sodium Lactate; CI confidence interval

* adjusted for patient to account for readmissions

^#^ XX have no outcome due to missing baseline Cl or Cl value in first 48 hours

^ p-value represents interaction term

**Table 4: Secondary outcomes as per intention-to-treat principle in the total trial cohort and subgroups.**

| **Characteristic** | **0.9% Sodium Chloride**  **N=** | **Plasmalyte-148**  **N=** | **Estimate of Difference (NS vs PL)*  (95% CI)** | **Compound Sodium Lactate**  **N=** | **Estimate of Difference (NS vs CSL)*  (95% CI)** |
| --- | --- | --- | --- | --- | --- |
| **Clinical outcomes** | | | | | |
| Survival free of organ dysfunction (days) *median (IQR)* |  |  |  |  |  |
| New onset AKI *n (%)* |  |  |  |  |  |
| Length of PICU stay (days) *median (IQR)* |  |  |  |  |  |
| Length of hospital stay (days) *median (IQR)* |  |  |  |  |  |
| PICU free survival (days) *median (IQR)* |  |  |  |  |  |
| **Safety outcomes** | | | | | |
| At least one adverse event *n (%)* |  |  |  |  |  |
| Hyponatremia *n (%)* |  |  |  |  |  |
| Hypercalcemia *n (%)* |  |  |  |  |  |
| Hypocalcemia *n (%)* |  |  |  |  |  |
| Hyperkalemia *n (%)* |  |  |  |  |  |
| Hypokalemia *n (%)* |  |  |  |  |  |
| Hypermagnesemia *n (%)* |  |  |  |  |  |
| Hyperlactemia *n (%)* |  |  |  |  |  |
| Death in hospital *n (%)* |  |  |  |  |  |

**Table 5: Protocol deviations**

| **Variable** | **Category** | **n** | **%** |
| --- | --- | --- | --- |
| Total number of protocol deviations reported | |  |  |
| Patients with at least one protocol deviation | |  |  |
| Number of protocol deviations per patient | 1 |  |  |
|  | 2 |  |  |
|  | 3 |  |  |
|  | ≥4 |  |  |
| Protocol deviation type | Patient received a different fluid from the one randomised to* |  |  |
|  | Time to obtain written consent >72 hours |  |  |
|  | Patient randomised but did not meet inclusion/exclusion criteria |  |  |
|  | Patient randomised but randomised fluid not commenced |  |  |
|  | Other |  |  |
| Protocol deviations in 0.9% Sodium Chloride arm | Number of maintenance fluid changes |  |  |
|  | Number of bolus fluid changes |  |  |
| Protocol deviations in Plasmalyte-148 arm | Number of maintenance fluid changes |  |  |
|  | Number of bolus fluid changes |  |  |
| Protocol deviations in Compound Sodium Lactate  arm | Number of maintenance fluid changes |  |  |
|  | Number of bolus fluid changes |  |  |

* 0.9% Sodium Chloride, Plasmalyte-48 or Compound Sodium Lactate not given as part of the intervention at a rate of >2ml/hour as well as 4% and 20% Albumin. Assessed from > 1 hour from randomisation to PICU discharge. Fluids that are considered as part of the intervention are: any fluid bolus and any IV maintenance. Total parenteral nutrition, enteral feeds and fluids, fluid used in drug dilution, fluid used as intravenous flush and blood products (packed red cells, fresh frozen plasma, cryoprecipitate) will not be considered for protocol violations. Each fluid not given as part of the intervention, that is given >1 hour apart will be considered a protocol violation.
