# Supplementary material for: 0.9% Sodium chloride solution versus Plasma-Lyte 148 versus compound sodium lacTate solution in children admitted to PICU—a randomized controlled trial (SPLYT-P): study protocol for an intravenous fluid therapy trial
Source: Trials. 2021 Jul 3;22:427. doi: 10.1186/s13063-021-05376-5 (PMC8254328; doi:10.1186/s13063-021-05376-5)
Supplement: Supplementary file 3 — Additional file 3. Parent/Guardian Information Sheet & Consent Form. [file 13063_2021_5376_MOESM3_ESM.docx]

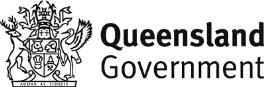


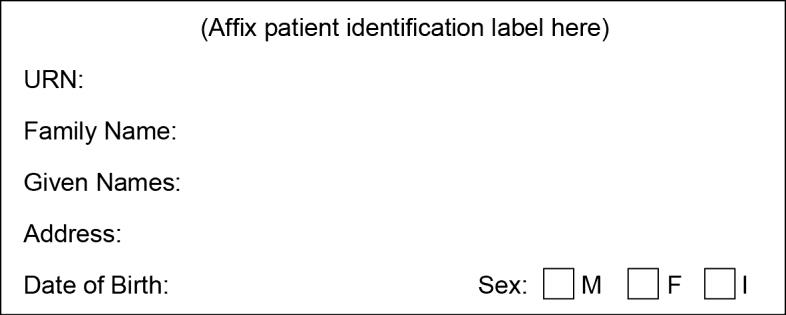
Children’s Health Queensland

Hospital and Health Service

**Clinical Research Consent**

**Parent/Guardian Information Sheet & Consent Form**

# Comparison of administration of 0.9% Sodium Chloride solution versus Plasma-Lyte 148 versus Compound Sodium LacTate Solution in children admitted to PICU – A randomised controlled trial

**It is okay to say no**

| **Local Principal Investigator** | | |
| --- | --- | --- |
| Dr xxxxxxx | xxxxx,  Queensland Children’s Hospital, Brisbane, Australia | xxxxxx via switch |

You and your child are being asked to participate in the comparison of administration of 0.9% sodium chloride solution versus Plasma-Lyte 148 versus Compound Sodium LacTate Solution in children admitted to Paediatric Intensive Care Unit (PICU) Study; and it is okay to say no if you do not want to be a part of this study. Your child has been admitted to the paediatric intensive care unit as part of their therapy in the hospital. This information sheet will tell you about this research study, explaining clearly and openly what is involved. This has been provided to help you decide whether or not you would like your child to take part in the research.

## Why is the study being done?

About 50,000 children are being admitted to hospitals across Queensland annually. Hospitalised children regularly receive intravenous fluids through a cannula placed on their hand or foot to keep them hydrated. The commonly used fluids for this therapy is called normal saline (0.9%NaCl). In some studies, in adult patients, this solution has been suspected as the cause of kidney dysfunction. There are two other fluids available which are often used. These are called balanced solutions (named Plasma-Lyte-148 and Compound Sodium Lactate) that may protect from this complication. From what is known, these three fluids currently used are safe. But we don`t know which of the fluids is the best one for children. In order to be sure which of these solutions is the best for hospitalised children, we need to do this study.

## What is the study about?

When children are unwell and admitted to hospital, they may not always be able to take food and drinks by mouth. Doctors support sick children in these instances by giving fluid (through a small cannula on the hand or foot) that contains necessary glucose, electrolytes and hydration. This study is assessing the use of three different intravenous fluids with varying composition of electrolytes. The commonly used solution is 0.9% NaCl. However, the composition of the two other crystalloids is closer to the human body (‘balanced solution’), namely PlasmaLyte-148 and Compound Sodium Lactate. As the composition of 0.9% sodium chloride and balanced solutions differ, the theoretical benefits and potential clinical side effects are also different.

## 0.9% NaCl Solution (also called normal saline)

0.9% NaCl solution represents the most common fluid used for intravenous fluids worldwide. Although it is called ‘normal’ saline, its composition can cause an increase in blood chloride level and make the blood more acidic. Usually the kidney help excrete this excess chloride from the body. However, when a child is unwell and dehydrated the kidneys may not work as well as usual. This can lead to a build-up of chloride in the body which may cause kidney injury.

## ‘Balanced’ Solutions - Plasma-Lyte 148 and Compound Sodium Lactate

Several studies observed better patient outcomes in adult patients treated with ‘Balanced’ solutions in comparison to 0.9% NaCl. The composition of ‘Balanced’ solutions is very similar to that of blood and fluid around body cells. Of note, the amount of chloride is considerably lower than that of 0.9% NaCl solution. The potential benefits of using ‘Balanced’ solutions for intravenous fluids compared to 0.9% NaCl are: a reduction in kidney injury, a probable reduction in the amount of inflammation in the body and perhaps better blood flow to the tissues. These effects could translate to a child recovering from their illness sooner and being discharged home earlier.

## What is the purpose of this research?

Currently, we do not know the best intravenous fluid solution for children. The purpose of this study is to investigate if treatment with a ‘Balanced’ solution (with reduced chloride content) is associated with less complications, faster recovery and improved outcomes of children who are admitted to the PICU and need intravenous fluids.

## How is the study being achieved?

We will randomly (by chance) allocate all children who are admitted to the PICU and need intravenous fluids (as decided by the doctor taking care of them) to one of the three fluid solutions.

Children in all three groups - 0.9% NaCl, Plasma-Lyte-148 and Compound Sodium Lactate - will follow the PICU protocols on best management. The doctor taking care of your child will decide: 1) if you child needs to receive Intravenous fluid, 2) how long to give and when to stop the intravenous fluid and 3) what quantity of fluid to give. Therefore, being part of this study will not change how your child is cared for on the PICU other than the type of fluid. All children in the study will be monitored carefully with constant nursing and medical staff presence.

Your child will require up to four blood samples of no more than 1 ml if they participate in this study. Often, doctors check blood electrolyte levels in all children who receive intravenous fluids. If your child is regularly having blood samples collected to check their electrolytes, no additional samples will be taken from your child as we are able to use these results for our study. However, if regular blood samples are not collected, we require samples at 4 time points. 1. Prior to commencement of the study 2. The morning after your child has been enrolled in the study. 3. Approximately 24 hours after enrolment. 4. Approximately 48 hours after enrolment or prior to fluids being stopped (whichever comes first) blood samples will be taken from arterial or venous lines that are already in place. In the event samples are unable to be collected from these lines, we will use capillary sampling which involves a skin prick and collection of a few drops of blood.

We will collect data from your child’s hospital records until they are discharged from hospital. Once your child stops receiving intravenous fluids or when they are discharged from PICU, their participation in the study will cease. No further follow-up will be necessary.

## Do I have to take part in this study?

Your child’s participation in this study is entirely voluntary. If you decide that you do not wish your child to take part in this study, your child will receive therapy based on the usual clinical practice of the doctors caring for them in the Intensive Care Unit.

## Are there any risks or side effects associated with the study?

Regardless of whether your child participates in this study or not, they are requiring intravenous fluids. The variation would be that they will receive one of the three fluids. Depending on the randomisation, this maybe 0.9% NaCl, Plasma-Lyte or Compound Sodium Lactate solution. All three fluids are already used in children for various scenarios where the doctor chooses to use them for their specific salt composition. Therefore, they pose no risks for harm to your child.

Where possible, we will avoid using the skin prick test. However, if required and your child is awake there maybe some discomfort with the skin prick sample. To minimise the discomfort from the skin pricks, we will offer to use topical local anaesthetic cream applied 30 minutes prior to the skin prick being performed.

Medical treatments - including intravenous fluids - can cause side effects. Your child may have none, some or all, of the effects listed below and they may be mild, moderate or severe. If your child has any of these side effects, or you are worried about them, please talk with their doctor. Your child’s doctor will also be looking out for any side effects.

Being in this study should not pose any additional risk to your child. Although these fluids have been given to many patients over many years and are in regular current use, there may be additional unforeseen or unknown risks.

***Potential*** *side effects of 0.9% NaCl include:*

- Increased blood chloride level and acidosis.
- Increased blood potassium level.
- Increased chance of having a kidney injury.
- Inflammatory response (tissues and blood vessels acting as if there was injury).

***Potential*** *side effects of Plasma-Lyte 148 include:*

- Decreased blood calcium level.
- Increased blood magnesium level.

***Potential*** *side effects of Compound Sodium Lactate include:*

- Increased blood calcium level.
- Decreased blood sodium level.

If at any point during the study your study doctor feels it is in your best interests not to continue receiving the study fluid; or if during the study there is evidence to suggest beyond reasonable doubt that the study fluid is not beneficial then your involvement in the study may be stopped.

## Are there any benefits associated with the study for my child?

This study may not have any direct benefits for your child. However, it will help us find out if any of the three fluid solutions compared in this study reduces complications, hospital length of stay and improves outcomes for children.

## Are there any benefits for other people in the future?

This study may not have any direct benefits for your child. However, it will help us find out if any of the three fluid solutions compared in this study reduces complications, hospital length of stay and improves outcomes for children.

## Will there be any costs for taking part in the study?

There will be no additional costs for participants in this study.

## Who will have access to the research records?

Nursing and medical staff in the Paediatric Intensive Care who are directly involved with this study will have access to the information collected. This study is occurring only at the Queensland Children’s’ Hospital and co- ordinated by the Paediatric Intensive Care Research Group at the Children’s Health Queensland Hospital and Health Service. The findings from the study will be anonymised and only available to the research team. Your child’s privacy will be maintained at all times. Your child’s name will not be used in any presentations or publications of the study results.

## What happens to data obtained through this study after the study is completed?

The de-identified study data will be kept for 15 years at the Centre for Children’s Health Research, which is a partnership between Children’s Health Queensland and the Mater Research Institute University of Queensland (MRI-UQ). Data thereby will be available for research. Any use of the data or samples will require approval by the Children’s Health Queensland, Human Research Ethics Committee.

## Withdrawal from the study

If you decide now, or at a later stage, that you do not wish your child to participate in this research project, that is entirely your right and will not in any way affect any present or future treatment.

If you decide not to participate in this study, this will in no way affect your current or future relationship with the hospital. It will also have no effect on your child’s treatment or your relationship with the people treating them. If you agree to your child participating in the study, you are free at any time to withdraw from the study without prejudice or reproach.

## Who do I speak to if any problems arise?

If you have any concerns about the way in which the research has been carried you, please do not hesitate to contact the Local Principal Investigator, which is outlined on the first page of this consent.

The Children’s Health Queensland Hospital and Health Service Human Research Ethics Committee (HREC) has approved this study. Should you wish to discuss the study with someone not directly involved, if any matters concerning policies, information about the conduct of the study or your rights as a participant, or you wish to make a confidential complaint, at any time, you may contact the Co-ordinator of the Ethics Committee on xxxx or email [xxxxx.](mailto:CHQETHICS@health.qld.gov.au)

***Thank you for your time and consideration of participation in this study.***


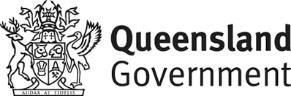


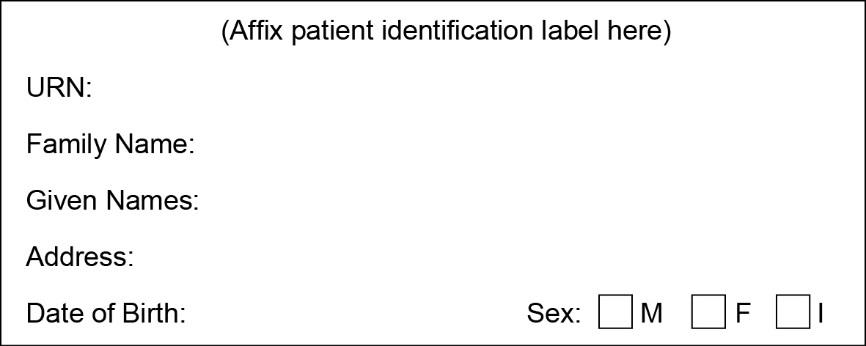
Children’s Health Queensland

Hospital and Health Service

**Clinical Research Consent**

**Parent/Guardian Consent Form**

# Comparison of administration of 0.9% Sodium Chloride solution versus Plasma-Lyte 148 versus Compound Sodium LacTate Solution in children admitted to PICU – A randomised controlled trial

- I understand that I have been asked to allow my child to participate in a study to investigate three intravenous fluid therapy solutions.
- I have read and understood the information sheet.
- The details of the study have been explained to me and my questions have been answered satisfactorily.
- The possible risks and benefits of my child participating have been explained to me.
- I understand that the project is for the purposes of research and not for treatment, so may not directly benefit me or my child.
- I have been informed that the confidentiality of the information will be maintained and safeguarded and give permission for

access to my child’s medical records for the purposes of research.

- I give permission for medical practitioners and other health professionals to release information concerning my child’s disease

and treatment which is needed for this trial and understand that such information will remain confidential.

- I understand that I may withdraw my child form the study at any time without affecting the care he/she receives.

## Comparison of the three fluid solutions

🞏 I agree to my child receiving the treatment allocation *in the Intensive Care Unit*.

**CONSENT**

I/We , being the parent(s)/guardian(s) of

give permission for my/our child to take part in this study. I/We would like to be informed of the study results **No** 🞏 **Yes** 🞏

If so please provide an email contact …………………………………………………………………………….

|  |  |  |
| --- | --- | --- |
| **Parent Name** | **Parent Signature** | **Date** |
|  |  |  |
| **Consenting Clinician Name** | **Consenting Clinician Signature** | **Date** |


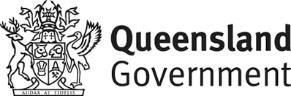


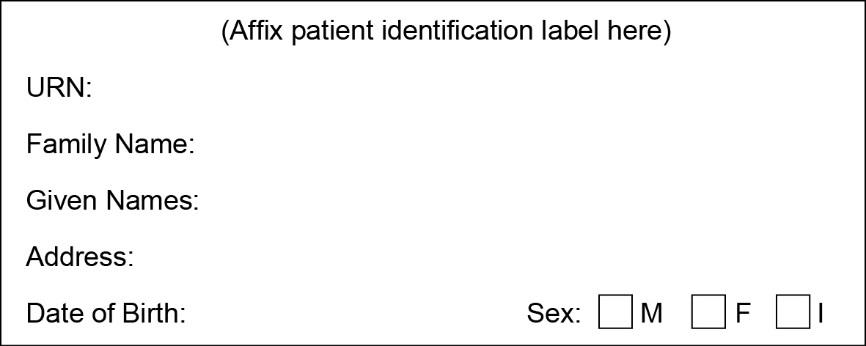
Children’s Health Queensland

Hospital and Health Service

**Clinical Research Consent**

**Parent/Guardian Withdrawal Form**

# Comparison of administration of 0.9% Sodium Chloride solution versus Plasma-Lyte 148 versus Compound Sodium LacTate Solution in children admitted to PICU – A randomised controlled trial

| **Local Principal Investigator** | | |
| --- | --- | --- |
| Dr xxxxx | xxxxxx,  Queensland Children’s Hospital, Brisbane, Australia | +xxxxxx via switch |

I hereby wish to **WITHDRAW** my intent for my child to participate further in the above research project and understand

that such withdrawal will not jeopardise my child’s future health care.

Child’s Name ……………………………………………………………..

Parent/Guardian Name ……………………………………………………………..

Parent/Guardian Signature …………………………………………………………….. Date…………………………

**If a verbal withdrawal:**

In the event the parent / guardian decided to withdraw verbally, please give a description of the circumstances. Principal Investigator to provide further information below:

Principal Investigator’s/Research Team Member Name …………………………………………

Principal Investigator’s/Research Team Member Signature ………………………………………… Date…………………

***Coordinating Investigator to sign the withdrawal of consent form on behalf of the parent / guardian if verbal withdrawal has been given***
